# Clinical efficacy of CO_2_ fractional laser in treating post‐burn hypertrophic scars in children: A meta‐analysis: CO_2_ fractional laser in treating post‐burn hypertrophic scars in children

**DOI:** 10.1111/srt.13605

**Published:** 2024-02-08

**Authors:** Yuan Chen, Wenlong Wei, Xiaojian Li

**Affiliations:** ^1^ Department of Burn and Plastic Surgery Guangzhou Red Cross Hospital Guangzhou China

**Keywords:** children, CO_2_ fractional laser, hypertrophic scars, post‐burn

## Abstract

**Objective:**

To evaluate and explore the efficacy of CO_2_ fractional laser in treating post‐burn hypertrophic scars in children through Meta‐analysis.

**Methods:**

English databases (PubMed, Web of Science and The National Library of Medicine), as well as Chinese databases (China National Knowledge Infrastructure and Wanfang Data) were searched. RevMan 5.3 software was used to data analysis.

**Results:**

A total of 10 pieces of literature were included, involving 413 children. Meta‐analysis showed that: (1) The average Vancouver Scar Scale after surgery was significantly lower than that before surgery [weight mean difference (WMD) = −3.56, 95% confidence interval (CI):−4.53,−2.58, *p* < 0.001]; (2) After CO_2_ fractional laser, pigmentation [WMD = −0.74, 95% CI:−1.10,−0.38, *p* < 0.001], pliability [WMD = −0.92, 95% CI:−1.20,−0.65, *p* < 0.001], vascularity [WMD = −0.77, 95% CI:−1.09,−0.46, *p* < 0.001], height [WMD = −0.57, 95% CI:−0.95,−0.19, *p* < 0.001] were improved compared with those before surgery. (3) The average Visual Analogue Scale (VAS) after surgery was significantly lower than that before surgery [WMD = −3.94, 95% CI:−5.69,−2.22, *p* < 0.001]. (4) Both Patient and Observer Scar Assessment Scale (POSAS)‐Observer [WMD = −3.98, 95% CI:−8.44,0.47, *p* < 0.001] and POSAS‐Patient [WMD = −4.98, 95% CI:−8.09,−1.87, *p* < 0.001] were significantly lower than those before surgery. (5) Erythema and vesicles were the most common complications after CO_2_ fractional laser therapy, with an incidence of 4.09%.

**Conclusion:**

CO_2_ fractional laser is beneficial to the recovery of hypertrophic scar after burn in children, and can effectively improve the scar symptoms and signs in children, with desirable clinical efficacy.

Hypertrophic scars (HS) are often secondary to burns, surgeries, lacerations, and so forth. The wounds after deep second‐degree burns reach deep into the dermis and require a long time to heal, resulting in a 50%−83% incidence of HS. In addition to exposing patients to varying degrees of pain, itching, dysfunction, and so forth, scars can also seriously affect their physical and mental health,^1^, ^2^ especially for children in the growth and development stage. Recently, there have been an increasing number of burn cases in children, which has seriously affected their psychological and physiological growth and development.^3^ It remains a challenge regarding the prevention and treatment of HS in burn patients. Traditional methods are unlikely to achieve the desired therapeutic effect, which is due to the unwillingness of children to cooperate with anti‐scar treatment, and the inability to maintain continuous pressure when applying pressure therapy in special parts of the treatment.

In the traditional treatment methods for scars, children have shown poorer compliance than adults during scar treatment methods. They are unwilling to actively cooperate with anti‐scar treatment, resulting in the inability to maintain sustained pressure on scars in joint areas. Therefore, traditional therapies have limited efficacy in treating HS in children.^4^ But all this has been revolutionized, a new scar treatment method that is, widely favored by doctors and scholars. Unlike traditional non‐surgical methods, laser technology boasts short treatment time, less trauma, quick recovery, easy operation, and favorable efficacy.^5^, ^6^, ^7^ Laser technology has been used in clinical applications for decades since the 1980s. Currently, various lasers such as fractional CO_2_ laser (CO_2_ fractional laser, CO_2_FL) and pulsed dye laser are available for scar treatment.^8^ There are differences in the efficacy of various lasers on scars. Many studies in recent years have revealed the role of CO_2_ fractional laser in wound repair, inhibiting excessive scar growth, and relieving pain and itching caused by scars.^9^, ^10^ Huang et al.^11^ showed that CO_2_ fractional laser can effectively treat HS and reduce pruritus caused by scars. Miletta et al.^12^ showed that, based on the theory of photothermal decomposition, a CO_2_ fractional laser with a wavelength of 10600 nm has achieved different degrees of efficacy in the treatment of scars. Tan et al.^13^ who used CO_2_ fractional laser to treat 221 patients with post‐burn HS and found that it can effectively improve the redness, swelling and hardness of scars.

Some studies have also reported on the efficacy of using CO_2_ fractional laser to treat post‐burn scars in larger areas and in younger children with varying degrees of severity. However, there are still inconsistencies in the research results. This study aimed to systematically collect relevant literature and conduct a meta‐analysis to explore the clinical effect of CO_2_ fractional laser in treating post‐burn HS in children.

## 1 MATERIALS AND METHODS

### 1.1 Literature search strategy

Following the guidelines of “Preferred Reporting Items for Systematic Reviews and Meta‐Analyses (PRISMA)”, English databases (PubMed, Web of Science and The National Library of Medicine), as well as the Chinese databases (China National Knowledge Infrastructure and Wanfang Data) were searched from their establishment to January 1, 2023. The English keywords were: “Lattice CO_2_ laser OR Fractional Carbon Dioxide Laser OR CO_2_ laser” AND “Scars OR Hypertrophic scar OR Cicatrix OR Hypertrophic Burn Scars ” AND “Burn” AND “Child OR Children OR Pediatric”.

### 1.2 Literature inclusion and exclusion criteria

Inclusion criteria: (1) Study type: retrospective studies, cohort studies and randomized controlled studies; (2) Study subjects: children with HS aged 3 to 16 who met the diagnostic criteria for HS in “Modern Scarology”^14^; (3) Interventions: CO_2_ fractional laser; (4) Outcome measures: preoperative and postoperative Vancouver Scar Scale (VSS) score, Visual Analogue Scale (VAS) score, The Patient and Observer Scar Assessment Scale (POSAS) score, adverse reactions, and so forth. Measurements at the last treatment session were extracted for all outcome indicators.

Exclusion criteria: (1) literature in which the participants received any treatment such as drug injection, radiotherapy, or surgery within 1 year; (2) literature with unclear description of relevant outcome indicators and unable to provide valid data; (3) literature without comparison data before and after treatment; (4) reviews, conference documents, expert speeches, and so forth; (5) animal experiments, repeated studies, case studies.

### 1.3 Literature screening and data extraction

Two investigators independently screened the literature. Initial screening was performed according to the title and abstract, and then secondary screening was performed by reading the full text according to the inclusion and exclusion criteria. The opinions of a third investigator were solicited and discussed to reach a consensus when there was disagreement. Data were extracted independently by two investigators.

### 1.4 Literature quality evaluation

The finally included cohort studies or retrospective studies were evaluated for quality in three aspects: selection, comparability, and exposure or outcome using the Newcastle–Ottawa Scale (NOS). A total of 9 points were set in this scale, and 1 point was scored if the scoring conditions were met. Low‐quality studies: scores <5, and high‐quality studies: scores ≥5. Studies with a NOS score <5 were not included.^15^

The quality of literature was assessed with reference to the Jadad Scale. Literature with a score ≥3 was considered to meet the inclusion criteria, and literature with a score <3 was considered to be of low quality^16^ and was not included. Literature that met the inclusion criteria was classified and assessed.

### 1.5 Statistical analysis

Revman5.3 software was used to meta‐analysis. The measurement data used the weight mean difference (WMD), while the count data used the relative risk as the effect indicator. Effect sizes were expressed as point estimates and 95% confidence intervals (CIs). For the heterogeneity test, $I^{2}$ test was used to judge the degree of heterogeneity, with $I^{2}\;$< 50% or *p* > 0.1 indicating the homogeneity of the included literature, which was analyzed using the fixed effects model (Mantel‐Haenszel). $I^{2}\;$> 50% or *p*≤0.1 indicated certain heterogeneity, which was analyzed using the random effects model (DerSimonian–Laird). The test level: *α* = 0.05.

## 2 RESULTS

### 2.1 Literature search results

**FIGURE 1:**
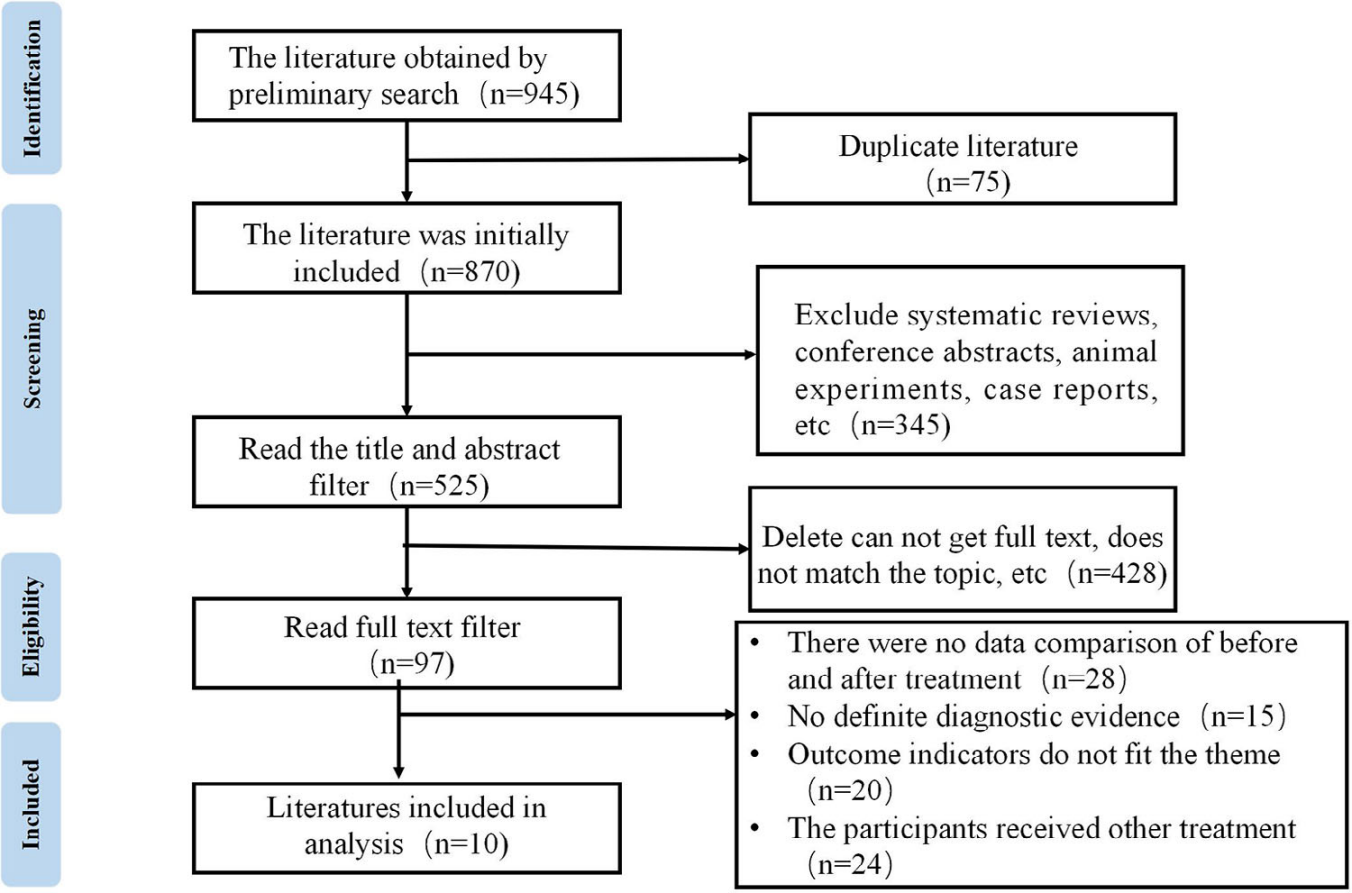
Flow chart of literature screening.

A total of 945 literature was retrieved through database retrieval, of which 75 replication studies as well as 345 systematic reviews, case reports and other studies were excluded. Studies without clear diagnostic criteria and no comparative data before and after treatment were excluded, and 10 pieces of literature were finally included for Meta‐analysis^17^, ^18^, ^19^, ^20^, ^21^, ^22^, ^23^, ^24^, ^25^, ^26^ (Figure 1).

### 2.2 Basic characteristics and quality assessment of the included literature

The 10 pieces of finally included literature were published from 2016 to 2023. The included literature involved a total of 425 children. The literature quality evaluation scores of the included retrospective studies and prospective cohort studies were between 6 and 8, both of which were medium‐high quality studies; The scores of the other two randomized controlled studies were both >3, which met the inclusion criteria. The basic characteristics and quality assessment results of the included literature are detailed in Table 1.

**TABLE 1:** Basic characteristics and quality assessment grades of the included literature.

Included literature	Year of publication	Country of publication	Study type	Sample size	Gender (male/female)	Age (years)	Pulse energy	Outcome indicators	Literature quality score
Julia Elrod^17^	2020	Switzerland	Retrospective study	17	8/9	11.37 ± 4.82	70–120 mJ	1,4	7
Sagar P. Patel^18^	2019	The United States	Prospective cohort study	49	26/23	4.86	13.7–101.6 mJ	3	8
Tomasz Za˛dkowski^19^	2016	Poland	Retrospective study	47	21/26	7.5 ± 2	60–150 mJ	1,4	8
Jennifer Zuccaro^20^	2018	Canada	Retrospective study	125	79/46	6.62 ± 5.36	53−78 mJ	1	7
Imran Majid^21^	2018	India	Retrospective study	10	3/7	9.7	90–150 mJ	1	8
TaeHo Won^22^	2022	China	Randomized controlled trial	20	13/7	2.35	30 mJ	3	5
Xing Fuxi^23^	2023	China	Retrospective study	43	23/20	2.9 ± 2.8	10–30 mJ	1,2,3,4	7
Zhou Nian^24^	2021	China	Retrospective study	45	–	3 ∼ 15	15–30 mJ	1	6
Xing Fuxi^25^	2021	China	Retrospective study	36	18/18	2.3 ± 0.2	50–100 mJ	1,2,4	6
Liu Jisong^26^	2023	China	Randomized controlled trial	33	25/8	2 ± 1.48	15–25 mJ	1,3,4	5

*Note*: 1 = Vancouver Scar Scale (VSS); 2 = Visual Analogue Scale(VAS); 3 = The Patient and Observer Scar Assessment Scale(POSAS); 4 = Adverse reactions.

### 2.3 Meta‐analysis results

#### 2.3.1 VSS

**FIGURE 2:**
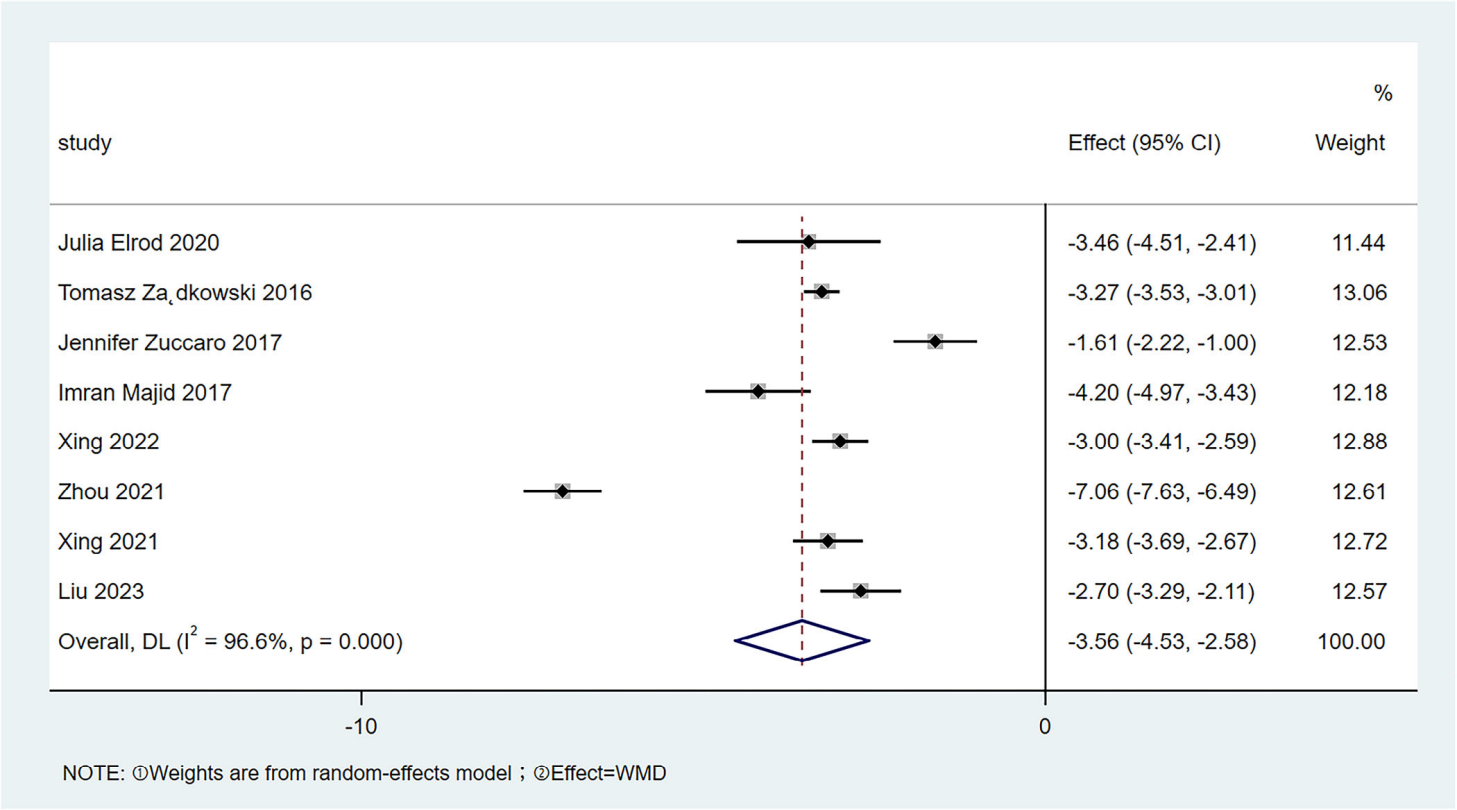
Forest plot of VSS differences. VSS, Vancouver Scar Scale.

Preoperative and postoperative data of VSS were reported in a total of eight pieces of literature. There was heterogeneity among the studies ( $I^{2}\;$= 96.6%) by meta‐analysis, so a random effects model was used, as shown in Figure 2. The results showed that the average VSS after surgery was lower than that before surgery [WMD = −3.56, 95% CI:−4.53,−2.58, *p* < 0.001].

#### 2.3.2 Pigmentation, pliability, vascularity, height

**FIGURE 3:**
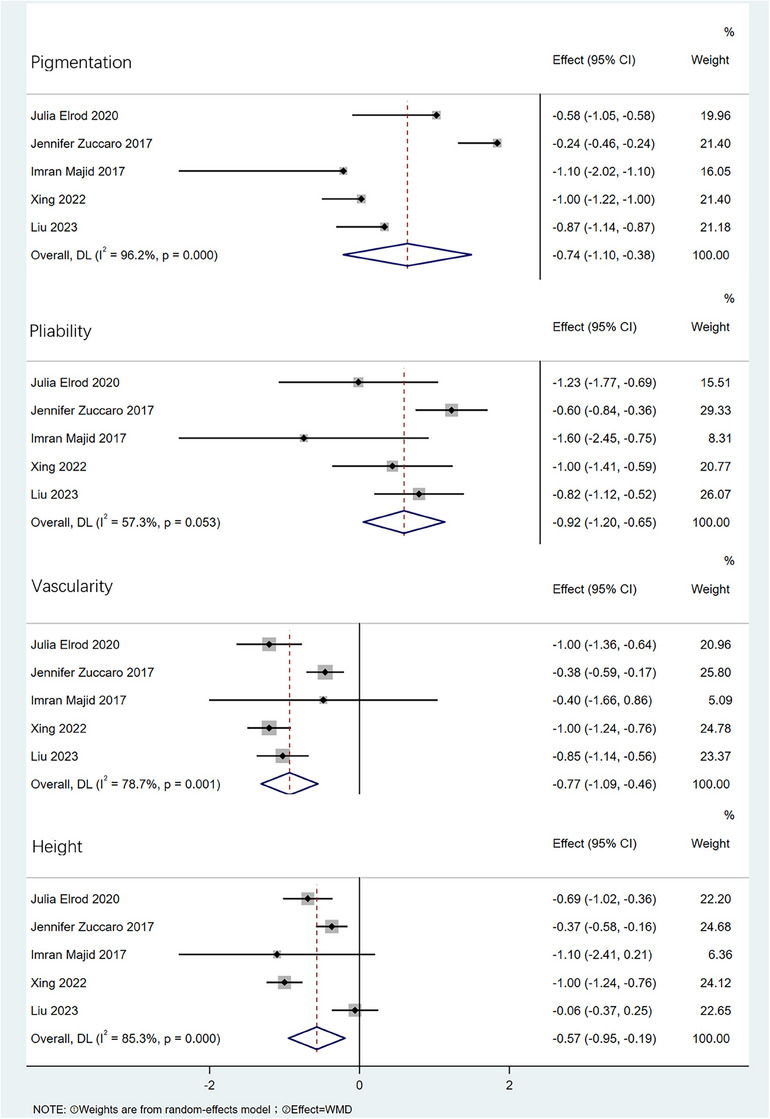
Forest plot of pigmentation, pliability, height, vascularity differences.

Preoperative and postoperative data on pigmentation, pliability, height, and vascularity were reported in a total of four pieces of literature. There was heterogeneity among the studies (pigmentation *I^2^* = 96.2%, pliability *I^2^* = 57.3%, vascularity *I^2^* = 78.7%, height *I^2^* = 85.3%) by meta‐analysis, so a random effects model was used, as shown in Figure 3. After CO_2_ fractional laser, pigmentation [WMD = −0.74, 95% CI:−1.10,−0.38, *p* < 0.001], pliability [WMD = −0.92, 95% CI:−1.20,−0.65, *p* < 0.001], vascularity [WMD = −0.77, 95% CI:−1.09,−0.46, *p* < 0.001], height [WMD = −0.57, 95% CI:−0.95,−0.19, *p* < 0.001] were improved compared with those before surgery, with statistically significant differences.

#### 2.3.3 VAS

**FIGURE 4:**
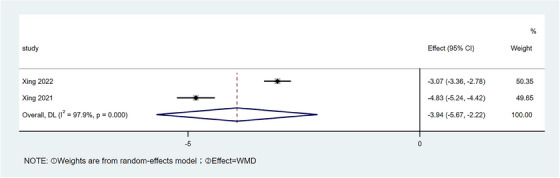
Forest plot of VAS differences. VAS, Visual Analogue Scale.

Preoperative and postoperative data of VAS were reported in a total of two pieces of literature. There was heterogeneity among the studies ( $I^{2}\;$= 97.9%) by meta‐analysis, so a random effects model was used, as shown in Figure 4. The results showed that the average VAS after surgery was lower than that before surgery [WMD = −3.94, 95% CI:−5.69,−2.22, *p* < 0.001].

#### 2.3.4 POSAS

**FIGURE 5:**
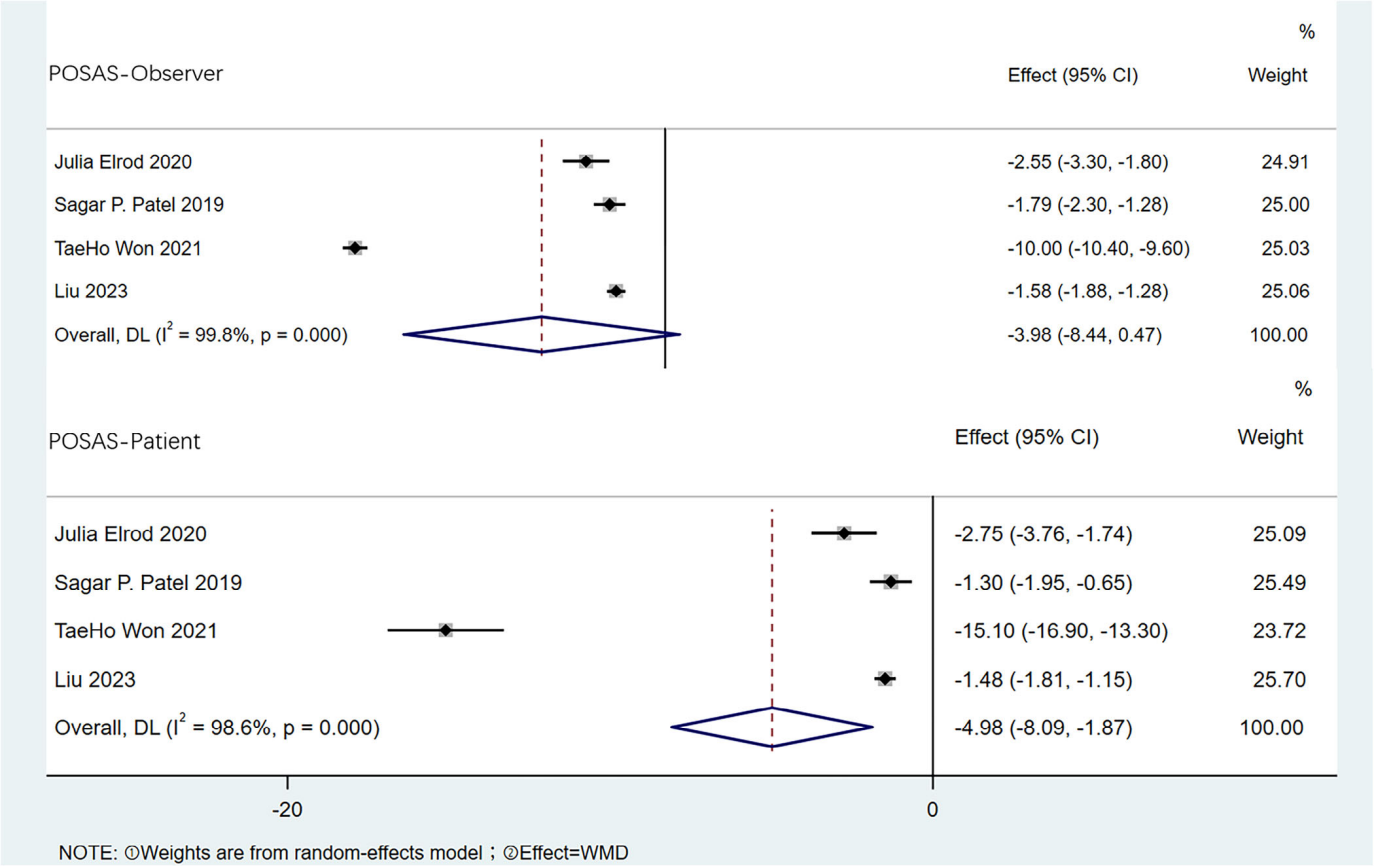
Forest plot of patient and Observer Scar Assessment Scale differences.

Postoperative and postoperative data on POSAS‐Observer and POSAS‐Patient were reported in a total of four pieces of literature. There was heterogeneity among the studies (POSAS‐Observer *I^2^* = 99.8%, POSAS‐Patient *I^2^* = 98.6%) by meta‐analysis, so a random effects model was used (Figure 5). The results showed that both POSAS‐Observer [WMD = −3.98, 95% CI:−8.44,0.47, *p* < 0.001] and POSAS‐Patient [WMD = −4.98, 95% CI:−8.09,−1.87, *p* < 0.001] were lower than those before CO_2_ fractional laser.

#### 2.3.5 Complications

The results of postoperative complications are shown in Table 2. The incidence of postoperative complications or adverse reactions was reported in a total of five pieces of literature. Among them, the most common postoperative complications were erythema and vesicle, with an incidence rate of 4.09%, followed by corrode, with an incidence rate of 3.51%. The incidence rates of discoloration, overgrowth and effusion of blood were all 2.34%. There were other rare complications, including wound infection and pigmentation, with an incidence of 1.17%.

**TABLE 2:** Incidence of postoperative complications.

Included literature	Sample Size	wound infection	erythema	Discoloration	Overgrowth	Effusion of blood	pigmentation	vesicle	corrode
Julia Elrod^17^	17	1	–	–	–	–	–	–	–
Tomasz Za˛dkowski^19^	47	–	2	4	4	–	–	–	–
Xing Fuxi^23^	43	–	–	–	–	–	1	7	5
Xing Fuxi^25^	31	1	3	–	–	4	–	–	–
Liu Jisong^26^	33	–	2	–	–	–	1	–	1
Incidence	–	1.17%	4.09%	2.34%	2.34%	2.34%	1.17%	4.09%	3.51%

## 3 DISCUSSION

In this study, based on the 10 pieces of included literature, this analysis showed that CO_2_ fractional laser improved the appearance and morphology of post‐burn HS in children through the preoperative and postoperative scales of VSS and POSAS. Moreover, CO_2_ fractional laser significantly relieved the degree of itch, as assessed by preoperative and postoperative VAS scales. CO_2_ fractional laser rarely causes complications, with damage being mild and tolerated. Most of the literature reported that complications and adverse reactions were greatly improved after CO_2_ fractional laser therapy^19^, ^23^, ^24^

It was shown that after CO_2_ fractional laser therapy, the VSS score of post‐burn HS in children was significantly improved compared with that before surgery, which was consistent with the conclusions of a previous meta‐analysis. Peng et al. reported that included 20 pieces of literature and reported the clinical efficacy and safety of CO_2_ fractional laser. CO_2_ dot matrix laser treatment improved the VSS score. They found that CO_2_ fractional laser improved the VSS score. However, there is still s single study that puts forward inconsistent views. Douglas et al. found that the VSS score after CO_2_ fractional laser therapy was not significantly different from that before surgery.^27^ The reason may be that the evaluation of VSS is subjective, and scars may be evaluated by different therapists due to the generally lengthy time span of treatment, thus having a greater impact on the results.^28^, ^29^ In addition, it is worth mentioning that the dose of laser therapy was not standardized in the literature included in this study. Currently, there are very few quantitative studies in the available literature that refer to laser treatment, and most of the studies determine the dose of treatment based on the subjective judgment of the treating physician, which allows for variations in the effectiveness of laser treatment. A recent study^30^ showed that the degree of temperature rise and thermal damage after laser treatment of hyperplastic scarring was directly related to the laser dose in terms of laser energy density, pulse width, and spot diameter, suggesting that this may be one of the reasons for the different conclusions in different studies.

The study also showed that the pruritus degree of post‐burn HS in children was significantly reduced after CO_2_ fractional laser therapy.^11^ Studies have shown that CO_2_ fractional laser is effective in the treatment of proliferative paralysis scars and the reduction of cancer itching caused by paralysis scars, thereby improving the quality of life of patients. In the traditional treatment methods for scars, children have shown poorer compliance than adults during scar treatment methods. They are unwilling to actively cooperate with anti‐scar treatment, resulting in the inability to maintain sustained pressure on scars in joint areas. Therefore, traditional therapies have limited efficacy in treating HS in children. In contrast, CO_2_ fractional laser therapy treats HS primarily through focal photothermal action. During therapy, “rectangular” microthermal damage zones can be created, each of which will form micropores at a certain depth on the skin surface. The heat from the micropores will stimulate the body to initiate the skin damage repair program, thereby promoting the reconstruction and regeneration of the full layer of skin. What's more, CO_2_ fractional laser can also block the blood vessels in the scar and promote the apoptosis of cells in the scar, so as to achieve the purpose of scar treatment. CO_2_ fractional laser produces a micro‐thermal damage zone with less damage to the epidermis than conventional laser methods, and shows better efficacy.^31^

As shown in this Meta‐analysis, the POSAS‐Observer and POSAS‐Patient scores with post‐burn HS in children after CO_2_ fractional laser therapy were lower than those before therapy. This shows that the vascular distribution, flexibility, surface area, scar thickness, pigmentation, convexity of scar, pain and pruritus of post‐burn HS in children are significantly improved from the perspective of both operators and patients’ families. Patel et al. reported that 49 children treated with CO_2_ laser and found that the total POSAS score increased from 89.6 ± 17.5 to 76.6 ± 16.8, with further improvement to 69.2 ± 14.8 at the last treatment.^18^ In the present study, complications and adverse reactions of post‐burn hyperplastic scars in children treated by CO_2_ fractional laser were summarized and analyzed. The most common postoperative complications were erythema and vesicle, while rare complications included wound infection and pigmentation.

Recently, there have been an increasing number of burn cases in children, which has seriously affected their psychological and physiological growth and development. It remains a challenge in burn patients. Traditional methods are unlikely to achieve the desired therapeutic effect, which is due to the unwillingness of children to cooperate with anti‐scar treatment, and the inability to maintain continuous pressure when applying pressure therapy in special parts of the treatment. To this end, it is urgent to find a new therapy. This study is a meta‐analysis evaluating the efficacy of CO_2_ fractional laser therapy for HS after lower limb burns in children, demonstrating that this therapy achieves good clinical efficacy, which is worthy of clinical promotion.

## CONFLICT OF INTEREST STATEMENT

None of the authors have any personal, financial, commercial, or academic conflicts of interest.

## CONSENT FOR PUBLICATION

Not applicable.

## Data Availability

All data generated or analyzed during this study are included in this published article.
